# Effect of two different digital construction techniques of mandibular implant-assisted overdentures on peri-implant bone loss and posterior ridge resorption: a 3-year prospective randomized clinical trial

**DOI:** 10.1186/s12903-025-06425-0

**Published:** 2025-07-02

**Authors:** Nermeen El Sayed El Khamisy, Radwa M.K. Emera, Thuraya Maher Elmanci, Marwa Ahmed Aboelez

**Affiliations:** Faculty of Dentistry, Mansoura University, Mansoura, Egypt

**Keywords:** Milled denture base, 3D-printing denture base, Posterior ridge resorption, Vertical bone loss

## Abstract

**Objectives:**

Traditional complete denture (CD) design and fabrication techniques need several clinical and laboratory steps. To improve the qualities of denture base material and get rid of all the problems that come with the conventional fabrication process, novel production methods were approved. In the field of denture manufacture, new materials and computer-aided technology have been explored as potential solutions. So, this study aimed to investigate the effect of two different digital construction techniques of implant-assisted overdentures on peri-implant and posterior mandibular bone resorptions by CBCT.

**Materials and methods:**

Twenty patients who received mandibular overdenture constructed by different construction techniques were classified equally and randomly into two groups (*n* = 10 per group): **Group M**: received milled mandibular overdenture opposed by maxillary complete denture. **Group P**: received 3D-printed mandibular overdenture opposed by maxillary complete denture. Peri-implant bone loss and posterior ridge resorption were assessed at baseline (T0), at 1 year(T1), and 3 years(T3) by superimposition of pre and post-treatment CBCT.The Shapiro-Wilk test was used to access data that was not normally distributed. Two distinct groups were compared using the Mann-Whitney test. The Wilcoxon signed-rank test was used to compare paired groups.

**Results:**

Regarding vertical bone loss(VBL), Group P recorded higher VBL in comparison to Group M, where the mean was 0.57 ± 0.13, and 0.52 ± 0.12respectively, at T1 and 0.66 ± 0.11, 0.60 ± 0.12 at T3. There was a statistically significant difference at different times of evaluation within the groups where (*P* < 0.001). Regarding posterior ridge resorption(PRR), Group P recorded higher PRR in comparison to Group M, where the mean was 381 ± 18.5, 333 ± 17.2 respectively at T1and 533 ± 24.9, 478.5 ± 12.3 at T3. There was a statistically significant difference at different times of evaluation within the groups where (*P* = 0.005).

**Conclusion:**

Regarding the preservation of peri-implant alveolar bone and posterior ridge bone, milled implant-assisted complete overdentures may have more favorable clinical outcomes compared to 3D printed implant overdentures in the digitally constructed mandibular overdenture bases retained by two implants.

**Clinical relevance:**

Both milled and 3D printed implant overdenture impression techniques can be used for the construction of CAD/CAM-implant retained overdenture base. However, in regarding the peri-implant alveolar bone and posterior ridge bone resorption, milled implant-assisted complete overdentures may have more favorable clinical outcomes compared to 3D printed implant overdentures retained by two implants.

**Clinical trial registry number:**

(No.-NCT06720389) (03/12/2024).

## Introduction

Even with advancements in edentulism dentistry, anatomical, physiological, or financial limitations keep conventional complete dentures (CDs) a realistic alternative [1]. Chewing difficulties, phonetics, atrophy of the tissue supporting the denture, incorrect interocclusal relationship between the upper and lower dentures, and psychological shame are the main problems faced by edentulous people wearing dentures [2]. Even though many patients expressed satisfaction with their complete dentures, some issues surfaced, particularly with the lower denture: insufficient support, stability, and retention; decreased chewing efficiency; and denture motion, which made mastication uncomfortable [3]. For completely edentulous patients, the following alternatives for therapy are available: traditional complete dentures, fixed prostheses supported by implants, and implant overdentures.

Traditional CD design and fabrication techniques need a number of clinical and laboratory steps. In addition, fracture, retention loss, poor aesthetics, and incorrect occlusal vertical dimension are among the problems associated with conventional CDs. In order to improve the qualities of poly methyl methacrylate (PMMA) material and get rid of all the problems that come with the fabrication process, novel production methods were approved. In the field of denture manufacture, new materials, and computer-aided technology have been explored as potential solutions [4–6].

With the use of CAD/CAM technology, denture bases may now be machined precisely and accurately. This technology offers numerous benefits for implant-assisted overdentures, including the ability to create a virtual model of the patient’s oral features, including the soft tissues and implants [7]. The digital model is then loaded into a milling machine, which meticulously grinds the denture foundation from a solid block of biocompatible material, such as polymethyl methacrylate (PMMA) or composite resin [8]. Because dental restorations vary anatomically, the milling machines mix burs of various sizes.

Models, dental crowns, inlays, onlays, and frameworks for dental restorations and implants are all made by layering materials together using computerized (3D) printing. This process is known as additive manufacturing or 3D printing. The materials and techniques utilized in additive manufacturing are used to categorize the process. The two most often used techniques in the dentistry sector are selective laser sintering (SLS) and stereolithography equipment (SLA) [9–11].

Moreover, there are many benefits of digital denture construction techniques as fewer appointments, improved fitness and strength of denture base, short length of manipulating the prosthesis, reduced possibility of infection due to microbial proliferation on denture surfaces, improvements in clinical research standardization for detachable prosthesis, simple denture replication and trial denture fabrication utilizing digitally saved information and excellent quality control carried out by technicians and clinicians [12].

One of the essential goals of any implant-assisted prosthesis is the preservation of the peri-implant-supporting structure. Thus, the most critical factor influencing implant success is preserving the integrity of the peri-implant bone and posterior ridge bone distal to the implants. Numerous factors, including surgical trauma, cortical bone thickness, the type and quantity of forces applied, patient characteristics, and the kind of prosthesis supported by the implant, could affect the height and density of the peri-implant bone [13]. Furthermore, functional pressure is among the most important etiologic factors in residual ridge resorption (RRR), which is strongly advocated by implant-retained overdentures (IROs) [14, 15]. Bite forces can increase significantly when two implants are used to hold dentures in place [16], In contrast to a conventional CD, this could lead to a more severe RRR in the posterior mandible distant to the implants [17].

There are many radiographic evaluation techniques for assisting bone height changes, including Cone Beam Computed Tomography (CBCT), which generates a three-dimensional picture volume and analyzes data using real volumetric methods using special software to reformat modified visualization of the anatomy with a low radiation exposure [18]. Additionally, it can rebuild the volume that was photographed in any virtual plane and offer precise bone measurements [19, 20]. Although there are several studies [21–23] have compared CAD/CAM and conventional fabrication techniques of the denture base from many aspects, the literature lacks clinical studies that compared between the milled and 3D printed construction techniques, most studies have compared between them are in vitro studies [24–29]. Furthermore, there is not enough clinical evidence on how CAD/CAM milled and 3D-printed implant-assisted complete overdentures alter bone height. Therefore, comparing VBL and posterior ridge resorption between CAD/CAM milled and 3D printed implant-assisted complete overdentures was the aim of this study. The first null hypothesis in this clinical experiment is that CAD/CAM milled and 3D printed implant-assisted overdentures will not differ in the VBL surrounding the implant. According to the second null hypothesis, there will be no differences in the posterior ridge resorption.

## Methods

### Study design

**Fig. 1:**
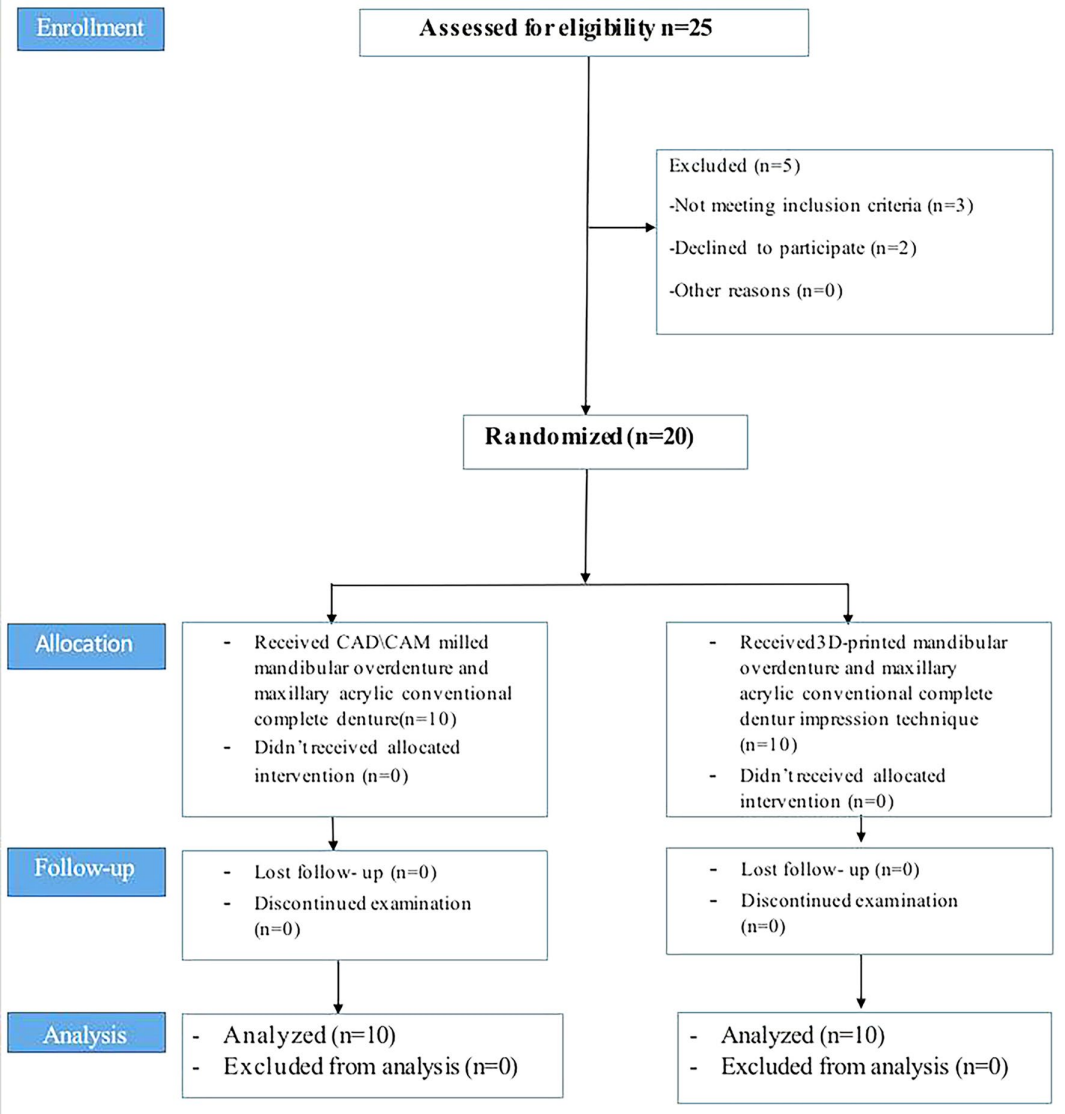
The study procedures’ flowchart

Twenty completely edentulous patients were selected from the outpatient clinic of the Removable Prosthodontic Department, Faculty of Dentistry, Mansoura University with ages between [55–70] years, and were included in the study (eight females and twelve males) in parallel randomized clinical trials. Figure 1 displays the study procedures’ flowchart. It was given the ethical approval number (A01010024RP). The study’s protocol was retrospectively registered in clinicaltrials.gov (No.-NCT06720389)(03/12/2024) Retrospectively.

### Data collection

The inclusion criteria involved patients with no evidence of inflammation or flappiness, and palpation with the blunt end of the mirror revealed that the maxillary and mandibular alveolar ridges were covered in a healthy, hard, reasonably even thickness, and even compressible mucosa. The preoperative 3D-panoramic analysis confirmed that the patients’ maxillary and mandibular ridges were completely edentulous, with a suitable mandibular bone height of at least 12 mm posteriorly and 15 mm or more in the interforaminal area. Based on preoperative tentative jaw relation data, all patients had adequate inter-arch space. Patients complained about the instability and lack of retention of their mandibular dentures. All patients were of Angel’s classI maxillo-mandibular relation. Patients who were definitively not qualified for implant placement due to conditions such as uncontrolled diabetes, osteoporosis, hyperparathyroidism, hematologic illnesses, liver conditions, and blood disorders, Patients with relative contraindications including smoking, alcoholism, and parafunctional habits (such as clenching and bruxism) were excluded.

### Sample size

After being briefed about the treatment protocols, patients gave their informed consent to take part. All experiment was conducted following all applicable rules and regulations. The G-power software (version 3.1.9.7) was used to determine the study’s sample size. The findings of the previous study [30] indicated that to achieve 95% power, and the effect size = 2, a sample size of 16 participants and 4 additional cases expecting the dropout (10 in each group) was used.

### Randomization and blinding

Based on the gender of the patient, participants were split into two groups; to guarantee an equal gender distribution between groups, twelve males and eight females were randomly selected for each group (*n* = 10 for each group). Using random numbers created in an Excel spreadsheet, the participants were assigned at random. Both groups are: (1) **Group M** (*n* = 10) received milled mandibular overdenture and maxillary acrylic conventional complete denture, and **Group P** (*n* = 10) received 3D-printed mandibular overdenture and maxillary acrylic complete denture. Individuals were randomly assigned to treatment groups by dental experts who were not aware of the treatment groups.

### Implant placement and prosthetic procedures

Conventional mandibular and maxillary dentures were made for both groups. Additionally, the scanning of the trial denture bases mounted on the articulator to register the vertical dimension and centric relation was done. To replicate the shape, form, and size of the teeth of the conventional denture in the new digitally constructed overdenture, the polished surface and occlusal relationship of the conventional maxillary and mandibular complete dentures were scanned using a 3D scanner (Medit I 500). Mandibular dentures were duplicated for each patient into heat-cure acrylic resin dentures for use as a radiographic template. On the buccal and palatal polished surfaces of the duplicate dentures, gutta-percha markings were placed. Using Cone Beam Computed Tomography (Vatech), two scans were taken for every patient [31]: one was taken while the patient was wearing the dentures, and the other was made for mandibular dentures). Using digital planning software (OnDemand3D, Cybermed Inc), the two scans were combined to create a single 3-dimensional image of the edentulous mandible. Using the software tools, the implants were virtually planned according to the correct angulation and distribution.

**Fig. 2:**
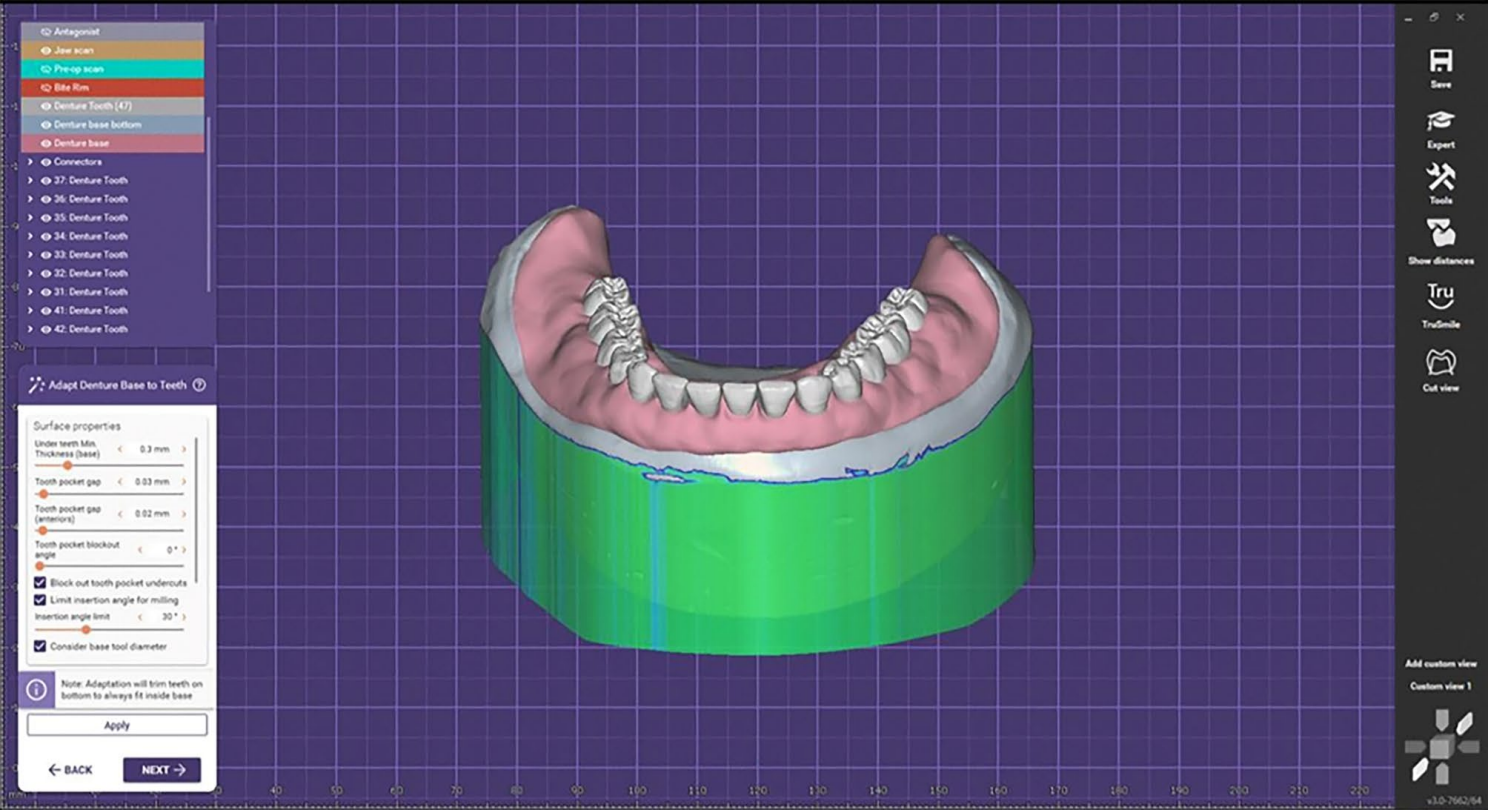
Virtual model for the mandibular permanent denture base

Two straight implants (11.5 mm long and 3.5 mm in diameter)(Neobiotech dental implant (IS-II active fixture)) were planned parallel to each other in the canine regions. A rapid prototyping machine (In2Guide) was used to create a mucosal-supported stereolithographic surgical guide with guided apertures for two implants that match the intended implant location and anchor pin placement. Using a flapless technique and delayed loading protocol, two implants (11.5 mm length and 3.5 mm diameter) were placed. Implant level impression using the open try impression technique was done for both groups according to E Jorge et al. [32]. The master cast was scanned extraorally with a 3D scanner (Shinning 3D) to obtain the STL file format of the virtual master cast.The jaw relation was scanned then all prostheses were constructed using a standardized bilateral balanced occlusion protocol, applied consistently across both groups. The virtual design of the mandibular permanent denture base, including the cavities of attachment housings with their escaping vents in the fitting surface, was approved. A digital preview was generated, verified, and approved before the fabrication of the final mandibular denture. A try-in step was then made with resin try-in of mandibular digital overdenture Fig. 2.

#### For group M: (Received milled mandibular overdenture)

**Fig. 3:**
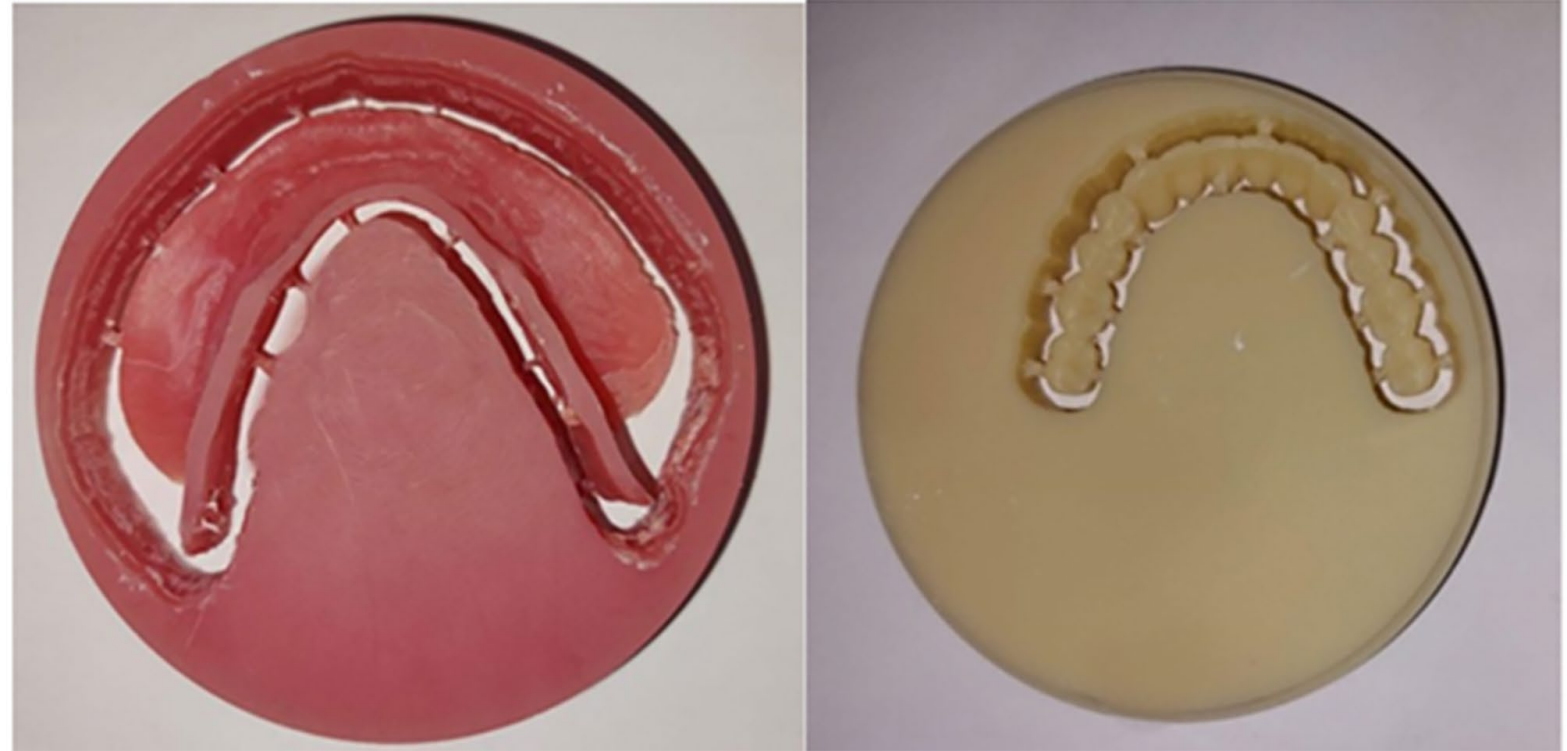
Milled mandibular denture base and teeth

Milled denture teeth from a block of tooth-colored PMMA (SR Vivodent CAD) and a denture base made from a pre-polymerized block of polymethylmethacrylate (Ivobase CAD) Fig.  3.

#### For group P: (Received 3D-Printed mandibular overdenture)

**Fig. 4:**
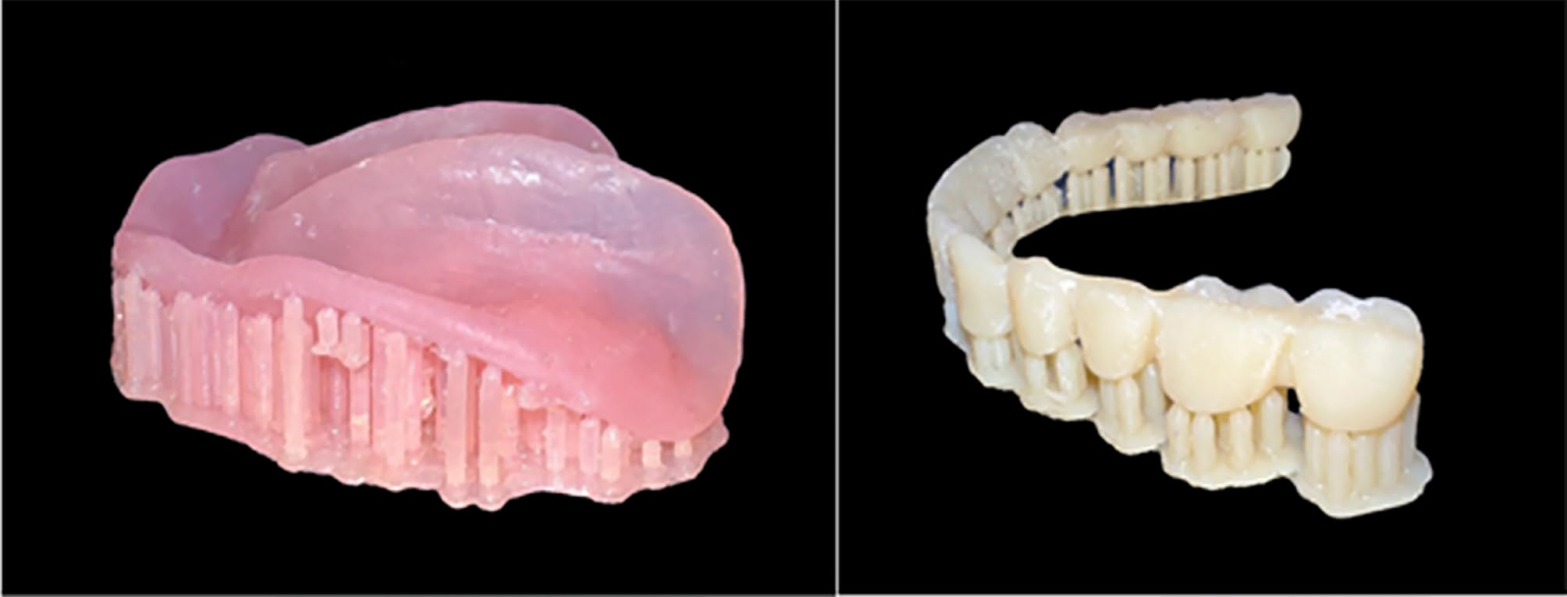
3D-Printed mandibular denture base and teeth

The printer (Rasdent 3d printer) was loaded with the material of 3D-printed denture base and teeth (Dentca denture teeth shade A2). The mandibular denture teeth were printed one unit and then bonded to the lower printed denture base Fig. 4.

**Fig. 5:**
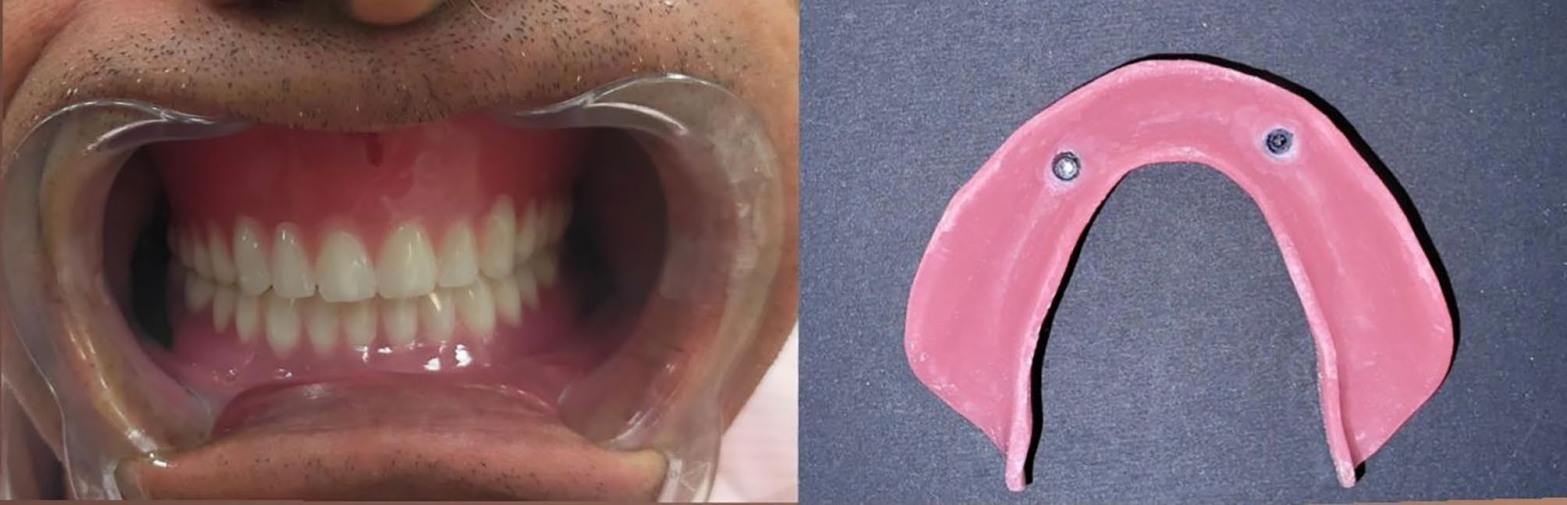
(**a**) Introral mandibular milled implant overdenture in occlusion with the maxillary conventional complete denture (**b**) Fitting surface of mandibular milled overdenture

**Fig. 6:**
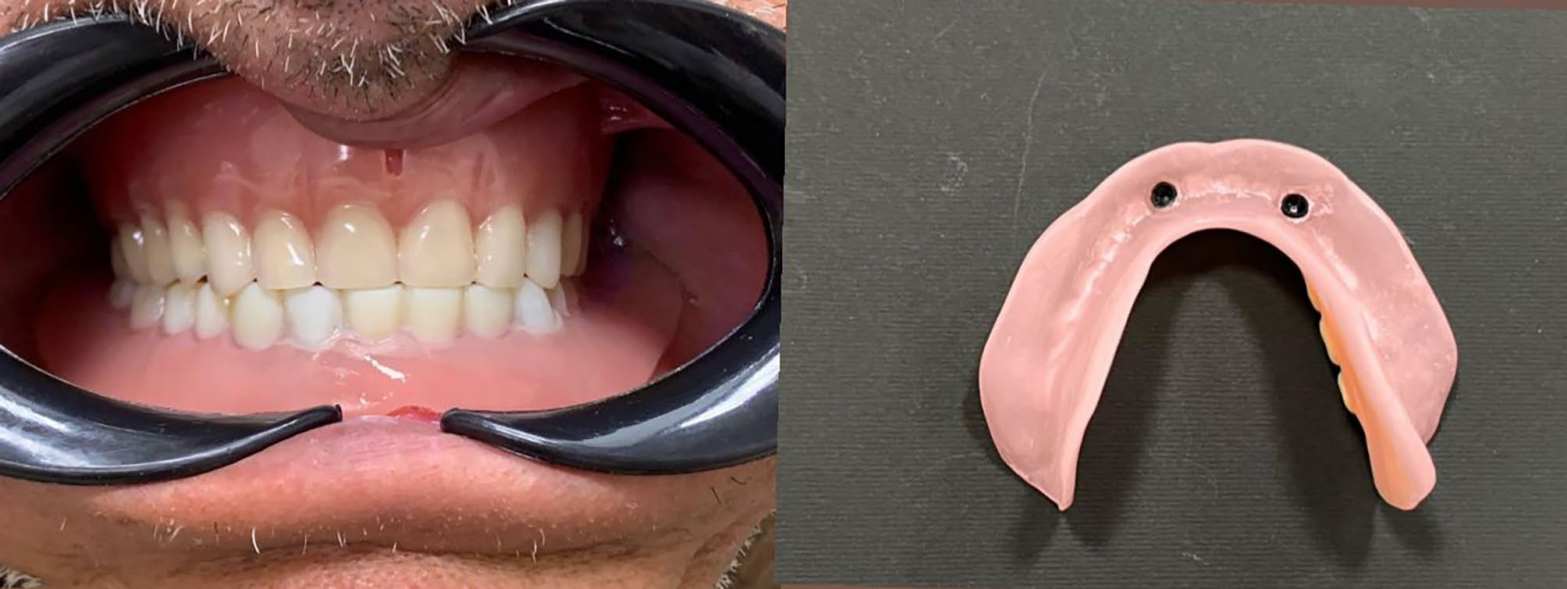
(**a**) Introral mandibular 3D printed implant overdenture in occlusion with the maxillary conventional complete denture (**b**) Fitting surface of mandibular 3D printed overdenture

For both groups: The attachments’ metal housings (Ball attachment) were placed extraorally into their cavities in the denture base’s fitting surface using Duralay.After removing any extra material, the denture base was finished and polished. The plastic retentive inserts were fitted into their housings, and the denture was inserted with ball attachments. The patient evaluated and approved the occlusion to correct any premature contacts or errors which may have an effect on bone resorption, esthetic, and phonetics of the prosthesis. Figs. 5 and 6.

Follow-up appointments were arranged for frequent data collecting following the delivery of the definitive prosthesis.

### Radiographic evaluation of the peri-implant bone level changes

The following scanning parameters were selected to obtain a high degree of measurement accuracy in the CBCT images: 120KvP, 5 mA, voxel size 0.25 mm, 14.7 s acquisition time, high-definition scan mode of 360° (total rotation), field of view (FOV diameter 16 cm), height 6 cm with a resolution of 0.157 × 0.157 mm. For quantitative and modeling analysis, the digital files of the images were obtained and saved. The digital imaging and communications in medicine (DICOM) files containing the three-dimensional volumetric images of each patient were exported and analyzed using image analysis software (Ondemand3D App v1.0.10.7510, CyberMed).

**Fig. 7:**
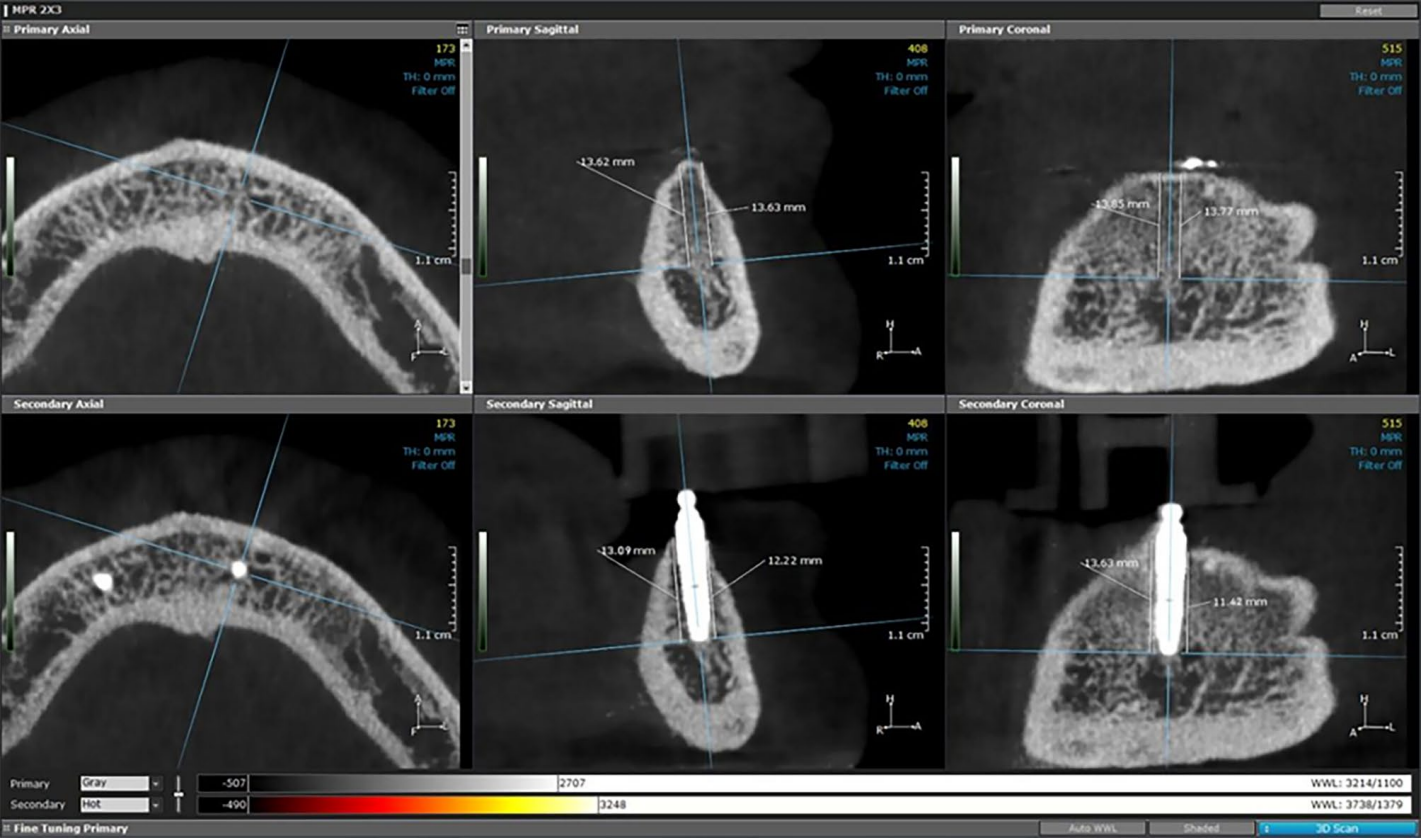
Measurements of peri-implant bone level changes by CBCT

Determine the baseline marginal bone level by identifying the long axis of the implant (central point of the apical and coronal parts of the implant), and create horizontal planes that are perpendicular to the implant’s long axis. The measures from the apical portion of the implant to the crest’s edge were the marginal bone level in millimeters. The four circumferential measurements were measured from the apical region of the implant to the edge of the crest (buccomesial, linguomesial, buccodistal, and linguodistal).Then, the marginal bone level at one, two and three years after insertion was determined and subtracted from the measures at the baseline [33] Fig.  7.

### Measurements of bone volume change of PRR

**Fig. 8:**
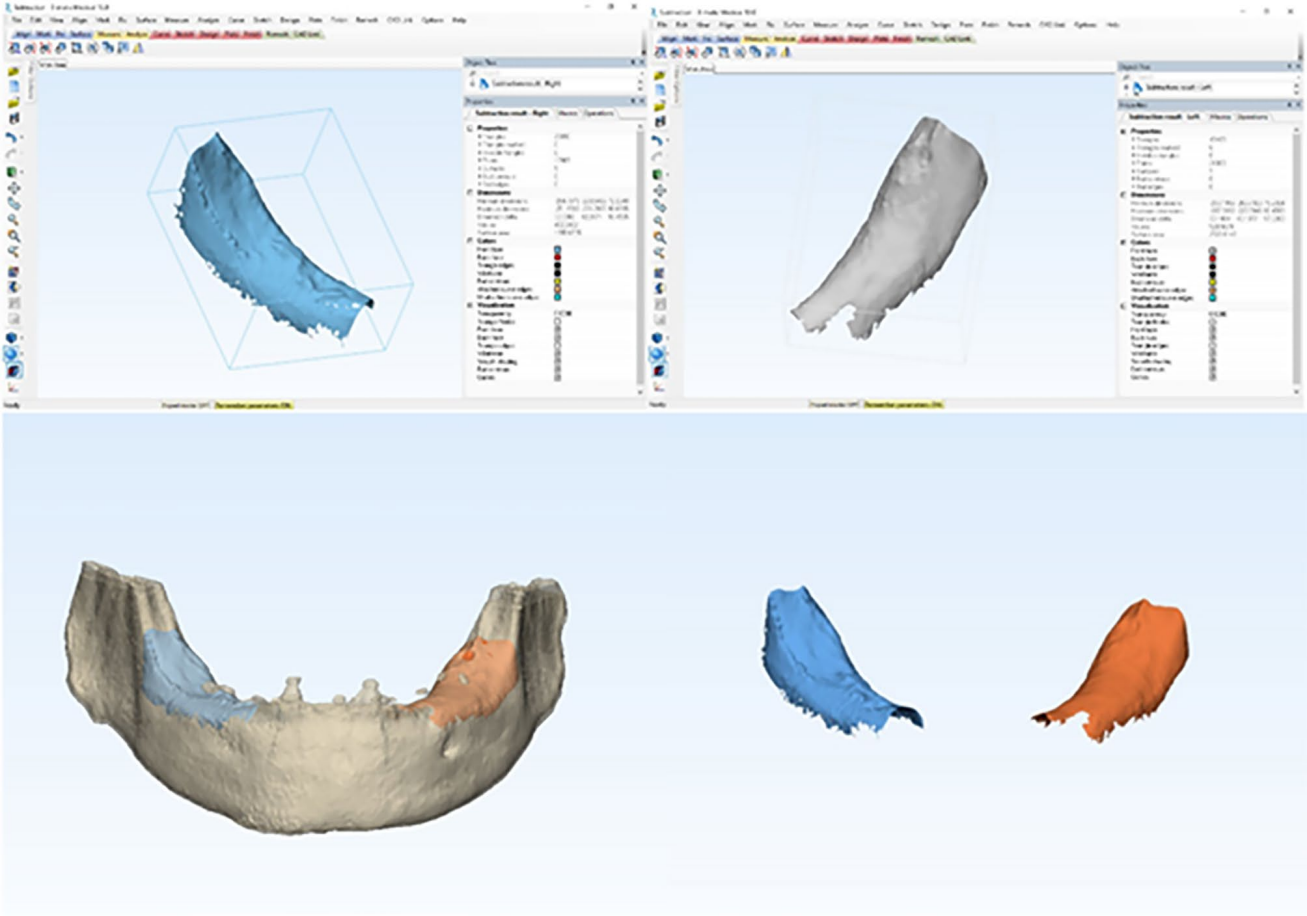
Volumetric measurements of bone change of PRR

One year post-treatment, the CBCT scans were superimposed on the diagnostic CBCT using Mimics version 14.1 (Materialise) and then exported into a different software application (3-matics version 5.1, Materialise). The changes in bone volume between preoperative CBCT and one to three years after insertion were used-in mm^3^ to quantify the PRR outcomes for both groups. To minimize the impact of CT artifacts, the focus region included the retromolar area immediately in front of the ascending ramus and approximately 5 mm distal to the implants at one, two and three years after insertion [34] Fig.  8.

### Statistical methods

The Statistical Package of Social Science (SPSS) software for Windows (Standard version 26) was used to analyze the data. The Shapiro-Wilk test was used to access data that was not normally distributed. Two distinct groups were compared using the Mann-Whitney test. The Wilcoxon signed-rank test was used to compare paired groups. The significance measure (p-value) is set at the 5% level. When *p* < 0.05, the results were considered significant. The results are more significant when the p-value is less.

## Results

Twenty participants were selected for this study, ages [55–70]. They were randomly divided into two groups. **Group M** received milled mandibular overdenture, and **Group P** received 3D-printed mandibular overdenture. Table 1 presents a comparison of the two groups’ variations in vertical bone loss (VBL). In the first year, there was no statistically significant difference in VBL between the groups (Mann-Whitney test, *p =* 0.427). Also, there was no significant difference between groups after 3 years (Mann Whitney test, *p* = 0.289).

**Table 1:** Comparison of VBL between groups

		Group M (*n* = 10)	Group P (*n* = 10)	Test of significance, *p*-value
T1	Mean ± SD	0.52 ± 0.12	0.57 ± 0.13	z = -0.795, *p* = 0.427
	Median	0.51	0.54	
	Min. to Max.	0.36 to 0.72	0.39 to 0.74	
T2	Mean ± SD	0.57 ± 0.12	0.62 ± 0.12	z = 1.252, *p* = 0.211
	Median	0.55	0.62	
	Min. to Max.	0.40 to 0.74	0.42 to 0.76	
T3	Mean ± SD	0.60 ± 0.12	0.66 ± 0.11	z = 1.059, *p* = 0.289
	Median	0.59	0.68	
	Min. to Max.	0.44 to 0.79	0.45 to 0.79	

Z: Mann-Whitney test, T1 at first year, T2at second year, T3 after 3 years

There was a statistically significant difference at different times of follow-up within all groups where (*P* = 0.005, Freidman test followed by several Wilcoxon signed ranks tests) in Table 2.

**Table 2:** Comparison of VBL with each group at different follow-up periods

	T1	T2	T3	Test of significance, *p*-value
Group M	0.51 (0.36 to 0.72)	0.55 (0.40 to 0.74)	0.59 (0.44 to 0.79)	Fr, *p* <0.001
				Z1= 2.816, *p* = 0.005
				Z2= 2.831, *p* = 0.005
				Z3= 2.831, *p* = 0.005
Group P	0.54 (0.39 to 0.74)	0.62 (0.42 to 0.76)	0.68 (0.45 to 0.79)	Fr, *p* <0.001
				Z1= 2.814, *p* = 0.005
				Z2= 2.807, *p* = 0.005
				Z3= 2.816, *p* = 0.005

Freidman test followed by several Wilcoxon signed ranks tests, Fr: Freidman test, z1: Wilcoxon signed ranks test between T 1, T 2; z2: Wilcoxon signed ranks test between T 1, T 3, z3: Wilcoxon signed ranks test between T 2, T 3where T1 at first year, T2at second year, T3 after 3 years

A comparison of posterior ridge resorption (PRR) between the studied groups is presented in Table 3. There were significant differences over the evaluation periods between groups, where (*P* < 0.001, Mann Whitney test).

**Table 3:** Comparison of PRR between groups

		Group M (*n* = 10)	Group P (*n* = 10)	Test of significance, *p*-value
T1	Mean ± SD	333 ± 17.2	381 ± 18.5	z = 3.722, *p* < 0.001
	Median	327.5	380	
	Min. to Max.	310 to 360	360 to 420	
T2	Mean ± SD	401.5 ± 11.1	428 ± 9.2	z = 3.700, *p* < 0.001
	Median	400	430	
	Min. to Max.	380 to 420	420 to 450	
T3	Mean ± SD	478.5 ± 12.3	533 ± 24.9	z = 3.756, *p* < 0.001
	Median	480	530	
	Min. to Max.	460 to 500	500 to 575	

Z: Mann-Whitney test, where T1 at first year, T2at second year, T3 after 3 years

Table 4 displays descriptive statistics of the variations in PRR between various follow-up periods within each group. In each group, there was a significant difference in PRR over time between each evaluation period where (*P* = 0.005, Freidman test followed by several Wilcoxon signed ranks tests).

**Table 4:** Comparison of PRR with each group at different follow-up periods

	T1	T2	T3	Test of significance, *p*-value
Group M	327.5 (310 to 360)	400 (380 to 420)	480 (460 to 500)	Fr, *p* <0.001
				Z1= 2.818, *p* = 0.005
				Z2= 2.807, *p* = 0.005
				Z3= 2.831, *p* = 0.005
Group P	380 (360 to 420)	430 (420 to 450)	530 (500 to 575)	Fr, *p* <0.001
				Z1= 2.836, *p* = 0.005
				Z2= 2.809, *p* = 0.005
				Z3= 2.818, *p* = 0.005

Freidman test followed by several Wilcoxon signed ranks tests, Fr: Freidman test, z1: Wilcoxon signed ranks test between T 1, T 2; z2: Wilcoxon signed ranks test between T 1, T 3, z3: Wilcoxon signed ranks test between T 2, T 3, where T1 at first year, T2at second year, T3 after 3 years

## Discussion

The purpose of this study was to assess the impact of two distinct digital construction techniques for mandibular implant-assisted overdentures on posterior mandibular ridge resorption and peri-implant resorption using CBCT. The first null hypothesis was accepted based on the study’s findings, which showed no significant difference in the VBL surrounding the implant between CAD/CAM milled and 3D-printed implant-assisted overdentures. However, the second null hypothesis was rejected as there was a statistically significant difference in posterior ridge resorption (PRR) between the two groups over all the observation periods.

CBCT was used in the current study to assess posterior ridge resorption changes and peri-implant bone variations. Volumetric data from CBCT is essential for the medical evaluation of the patient’s anatomy and any bone abnormalities. It offers a precise 3D image with less radiation exposure than traditional CT systems [35, 36].

VBL around implants in the first year was in reasonable ranges, with VBL not exceeding 0.8 mm. According to Albrektsson et al. [37], these results were consistent with their success criteria for implants, which were as follows: (a) annual bone loss should not surpass 0.2 mm, and (b) marginal VBL within the first year should be below 1.5 mm.

Moreover, no significant difference in VBL was found between the two groups during all the observation periods. It could be due to similar bone remodeling rates and the use of the same implant support and retentive inserts. This consistency was supported by Yoda et al. [38], who highlighted the relationship between attachment types and load distribution on implants.

In this investigation, there was a statistically significant difference in the vertical bone loss within each group through all the observation periods. One possible explanation for this could be that elderly people are less physically agile, which leads to less cleaning. As a result, additional plaque was gradually deposited, causing gingival irritation and an increased deposition of more plaque, which led to increased VBL around implants.

In contrast, Turkyilmaz et al. [39] demonstrated a negligible rise in these parameters over time. Furthermore, it seems that the patients who were part of this study did not practice good oral hygiene. It should be emphasized that the results of VBL scores could be altered if a dental hygienist followed and monitored a strict cleaning and hygiene schedule. In contrast, Aboelez et al. [40] found that ball attachments were associated with lower plaque, gingival scores, VBL, and higher patient satisfaction with cleaning ease.

On the other hand, there was a remarkable increase in the VBL in group **P** than in group **M**, which might be because the milled group showed significantly higher whole surface area adaptation than the printed group, which allows better stress distribution around the implants, causing less VBL resorption. This is consistent with the results of Faty et al. [41], who evaluated the retention and adaptability of 3D-printed and milled denture bases and contrasted them with conventional ones. The findings revealed notable variations among the three groups, with the milled group exhibiting the highest levels of denture base adaption and retention.

After three years of loading, there was a significant decrease in posterior ridge resorption (PRR) in group **M** than in group **P**. This might be because milled dentures provided significantly better retention and adaptation than 3D-printed dentures. These findings were in line with another study [42] which found that the superior fit of CAD-CAM milled dentures was attributed to the unique manufacturing approach because volumetric variations related to denture base processing are eliminated since the denture base is milled from a fully pre-polymerized resin puck that was polymerized at high temperature and pressure using a subtractive approach.

Regarding the 3D-printed denture bases, an un-polymerized resin was utilized in the fabrication of the denture base so that polymerization shrinkage is hypothetically viable, as the denture bases are not completely polymerized before the last light-polymerization process [43].

Moreover, the stair-stepping phenomena in 3D printing show that surface roughness, a key component of plaque accumulation and bacterial adherence, is greatly influenced by the thickness of each layer. These findings were in line with another study [44], which found that a lower degree of polymerization and substantial monomer leakage may have contributed to the 3D-printed specimens’ enhanced roughness by raising surface porosity and roughness. Another study [45]found that in contrast to the smoother surfaces of the milled specimens, the 3D-printed specimens had higher surface roughness values.

On the other hand, previous studies compared three distinct methods of denture base fabrication: conventional, milled, and 3D printed, and found that the 3D-printed denture bases had better tissue adaption [46–48]. Interestingly, they found that 3D-printed dentures produced better results in terms of force distribution and occlusal force. Furthermore, digitally fabricated dentures were reported to preserve occlusion more effectively than conventionally fabricated ones by Chaturvedi et al. [49].

In summary, in terms of bone height changes, milled and 3D printed implant-assisted complete overdenture bases showed no significant difference in vertical bone loss; however, milled implant-assisted complete overdentures showed more favourable results than 3D printed implant overdentures for posterior ridge resorption.

One of the study’s limitations is the small sample size of the patient population that was used. Further research with a larger sample size and consideration of additional confounding factors is required because they are also one of the study’s limitations.

## Conclusion

Regarding the preservation of peri-implant alveolar bone and posterior ridge bone, milled implant-assisted complete overdentures may have had more favourable clinical outcomes compared to 3D printed implant overdentures in the digitally constructed mandibular overdenture bases retained by two implants.

## Data Availability

The datasets used in the current study are available from the corresponding author upon request.

## Abbreviations

CD: Complete denture
PMMA: Poly methyl methacrylate
SLS: Selective laser sintering
RRR: Residual ridge resorption
IROs: Implant-retained overdentures
CBCT: Cone beam computed tomography
DICOM: Digital imaging and communications in medicine
SPSS: Statistical package of social science
VBL: Vertical bone loss
PRR: Posterior ridge resorption

## References

[1] Janeva NM, Kovacevska G, Elencevski S, Panchevska S, Mijoska A, Lazarevska B. Advantages of CAD/CAM versus conventional complete dentures- a review. Open Access Maced J Med Sci.2018;6(8):1498–502. 10.3889/oamjms.2018.308. eCollection 2018 Aug 20. 10.3889/oamjms.2018.308 PMC6108805 30159084

[2] Superior CDE, Silva AS, Aroso C, Superior CDE. Juliana De SÁ EYWORDS.2017; 3–8.

[3] Gray D, Patel J. Implant-supported overdentures: part 1. Br Dent J. 2021;231(2):94–100. 10.1038/s41415-021-3224-4. 34302089 10.1038/s41415-021-3224-4

[4] Perea-Lowery L, Minja IK, Lassila L, Ramakrishnaiah R, Vallittu PK. Assessment of CAD-CAM polymers for digitally fabricated complete dentures. J Prosthet Dent. 2021;125(1):175–81. 10.1038/s41415-021-3224-4. 32063383 10.1016/j.prosdent.2019.12.008

[5] Lee S, Hong SJ, Paek J, Pae A, Kwon KR, Noh K. Comparing accuracy of denture bases fabricated by injection molding, CAD/CAM milling, and rapid prototyping method. J Adv Prosthodont. 2019;11(1):55–64. 10.4047/jap.2019.11.1.55. 30847050 10.4047/jap.2019.11.1.55 PMC6400705

[6] Mathew T, Kattadiyil CJ, Goodacre, Nadim ZB. Complete dentures: of two commercial fabrication systems. CDA J. 2013;41(6):407–16. 23875432

[7] Mühlemann S, Hjerppe J, Hämmerle CH, Thoma DS. Production time, effectiveness and costs of additive and subtractive computer-aided manufacturing (CAM) of implant prostheses: A systematic review. Clin Oral Implants Res. 2021;32(211):289–302. 10.1111/clr.13801. 34642980 10.1111/clr.13801 PMC9293467

[8] Basunbul AI. Analysis of the mechanical and physical properties of printed and milled denture base materials [thesis].2021 Boston: Boston University.

[9] Barazanchi A, Li KC, Al-Amleh B, Lyons K, Waddell JN. Additive Technology: Update on Current Materials and Applications in Dentistry. J Prosthodont.2017;26(2):156–63. 10.1111/jopr.12510 Available from: www.wileyhealthlearning.com/jopr 10.1111/jopr.12510 27662423

[10] Bae EJ, Jeong I, Do, Kim WC, Kim JH. A comparative study of additive and subtractive manufacturing for dental restorations. J Prosthet Dent. 2017;118(2):187–93. 10.1016/j.prosdent.2016.11.004. 28089336 10.1016/j.prosdent.2016.11.004

[11] Rath AA. Review edentulism in elderly: a review of current clinical concerns in India. J Geriatr Care Res. 2018;5(1):22–7.

[12] Bidra AS, Taylor TD, Agar JR. Computer-aided technology for fabricating complete dentures: systematic review of historical background, current status, and future perspectives. J Prosthet Dent. 2013;109:361–6. 10.1016/S0022-3913(13)60318-2. 23763779 10.1016/S0022-3913(13)60318-2

[13] Tsigarida A, Chochlidakis K, Fraser D, Lampraki E, Einarsdottir ER, Barmak AB. Peri-implant diseases and biologic complications at implant-supported fixed dental prostheses in partially edentulous patients. J Prosthodont.2020;29:429– 35. 10.1111/jopr.13165. Epub 2020 May 6. 10.1111/jopr.13165 32180293

[14] Canger EM, Çelenk P. Radiographic evaluation of alveolar ridge heights of dentate and edentulous patients. Gerodont 29:17–23. 10.1111/j.1741-2358.2010.00391.x 10.1111/j.1741-2358.2010.00391.x 20545771

[15] Blum IR, McCord JF. (2004) A clinical investigation of the morphological changes in the posterior mandible when implant-retained overdentures are used. Clin Oral Implants Res. 2004;15:700–708. 10.1111/j.1600-0501.2004.01057.x 10.1111/j.1600-0501.2004.01057.x 15533131

[16] Fontijn-Tekamp FA, Slagter AP. Biting and chewing in over- dentures, full dentures, and natural dentitions. J Dent Res. 2000;79:1519–24. 10.1177/00220345000790071501. 11005738 10.1177/00220345000790071501

[17] Slot W, Raghoebar GM, Vissink A, Meijer HJ. A comparison between 4 and 6 implants in the maxillary posterior region to support an overdenture; 1-year results from a randomized controlled trial. Clin Oral Implants Res. 2014;25(5):560–6. 10.1111/clr.12118. 23406268 10.1111/clr.12118

[18] Nomier AS, Gaweesh YSE, Taalab MR, El Sadat SA. Efficacy of low-dose cone beam computed tomography and metal artifact reduction tool for assessment of peri-implant bone defects: an in vitro study. BMC Oral Health 2022;17;22(1):615. 10.1186/s12903-022-02663-8.PMID: 36528573. 10.1186/s12903-022-02663-8 PMC9759909 36528573

[19] Jacobs R, Salmon B, Codari M, Hassan B, Bornstein MM. Cone beam computed tomography in implant dentistry: recommendations for clinical use. BMC Oral Health. 2018;18:88. 10.1186/s12903-018-0523-5. 29764458 10.1186/s12903-018-0523-5 PMC5952365

[20] Mourad KE, Emera RMK, Ahmed W, Habib A. Lateral incisors versus canine areas for two implant placements used to retain mandibular overdenture: periodic monitoring of ridge base contact relation. J Dent Implants. 2020;10(2):72.

[21] Faty MA, Sabet ME, Thabet YG. A comparison of denture base retention and adaptation between CAD/CAM and conventional fabrication techniques. Int J Prosthodont. 2023;36(4). 10.11607/ijp.719. 10.11607/ijp.7193 37699188

[22] Khorasani E, Mokhlesi A, Arzani S, Ghodsi S, Mosaddad SA. Are There Clinical Differences Between 3D-Printed and Milled Complete Dentures? A Systematic Review and Meta-analysis. Int Dent J.2024;12:S0020-6539(24)01593-4. 10.1016/j.identj.2024.11.007 10.1016/j.identj.2024.11.007 PMC11976578 39672779

[23] Chebib N, Imamura Y, El Osta N, Srinivasan M, Müller F, Maniewicz S. Fit and retention of complete denture bases: part II - conventional impressions versus digital scans: A clinical controlled crossover study. J Prosthet Dent. 2024;131(4):618–25. Epub 2022 Aug 30. PMID: 36055812. 36055812 10.1016/j.prosdent.2022.07.004

[24] El Samahy MM, Abdelhamid AM, El Shabrawy SM, Hanno KI. Evaluation of physicomechanical properties of milled versus 3D-printed denture base resins: A comparative in vitro study. J Prosthet Dent. 2023;129(5):797.e1-797.e7. doi:10.1016/j.prosdent.2023.03.017. PMID: 37121625. 10.1016/j.prosdent.2023.03.017 37121625

[25] Yoon HI, Hwang HJ, Ohkubo C, Han JS, Park EJ. Evaluation of the trueness and tissue surface adaptation of CAD-CAM mandibular denture bases manufactured using digital light processing. J Prosthet Dent. 2018;120(6):919–26. 10.1016/j.prosden. 29961610 10.1016/j.prosdent.2018.01.027

[26] Hwang SJL, Park EJ, Yoon HI. Assessment of the trueness and tissue surface adaptation of CAD-CAM maxillary denture bases manufactured using digital light processing. J Prosthet Dent. 2019;121(1):110–7. 10.1016/j.prosdent.2018.02.018. 30006217 10.1016/j.prosdent.2018.02.018

[27] Goodacre CJG, Baba NZ, Kattadiyil MT. Comparison of denture base adaptation between CAD-CAM and conventional fabrication techniques. J Prosth Dent. 2016;116(2):249–56. 10.1016/j.prosdent.2016.02.017. 10.1016/j.prosdent.2016.02.017 27112416

[28] Steinmass HD, Grunert I, Steinmass PA. CAD/CAM produces dentures with improved fit. Clin Oral Invest. 2018;22(8):2829–35. 10.1007/s00784-018-2369-2. 10.1007/s00784-018-2369-2 29468600

[29] Peng LC, Harris PT, Bhandari B, Morton D, Lin. WS. Accuracy and reproducibility of virtual edentulous casts created by laboratory impression scan protocols, J Prosth Dent. 2018;120(3): 389–395. 10.1016/j.prosdent.2017.11.024 10.1016/j.prosdent.2017.11.024 29703675

[30] Elmahdy AA, Eid HI, Ouda SL. Effect of two different types of denture base materials on the supporting structures of mandibular Mini implant supported over denture. ASDJ. 2021;24:112–18. 10.21608/asdj.2021.59640.1016.

[31] De Kok IJ, Thalji G, Bryington M, Cooper LF. Radiographic stents: integrating treatment planning and implant placement. Dent Clin North Am. 2014;58(1):181–92. 10.1016/j.cden.2013.09.008. 24286652 10.1016/j.cden.2013.09.008

[32] Jorge E, Ercoli FPD, Moss C, Graser ME, Tallents GN. Verification jig for implant-supported prostheses: A comparison of standard impressions with verification jigs made of different materials. J Prosthet Dent Int J Oral Maxillofac Implants. 2002;88(3):329–36. 10.1067/mpr.2002.128070. 10.1067/mpr.2002.128070 12426505

[33] Almayah HBM, Hassan TA, Al-Jumaily HA, Al-Ghurabi ZH. Evaluation of marginal bone loss around SLActive implants by CBCT using different implant dimensions and surgical approaches: A clinical and radiological prospective study. J Osseointegr. 2022;14:1–5. 10.23805/JO.2021.14.1.

[34] Ahmad R, Chen J, Abu-Hassan M, Li Q, Swain MV. Investigation of Mucosa-Induced residual ridge resorption under Implant-Retained overdentures and complete dentures in the mandible. Int J Oral Maxillofac Implants. 2015;30:657–66. 0.11607/jomi.3844. 26009917 10.11607/jomi.3844

[35] Torresin A, de las Heras Gala H, Dasu A, Andersson J, Caprile P, Darréon J. SP-0689: CBCT QA: European guidelines by EFOMP-ESTRO-IAEA. Radiother Oncol 127:S360–1. 10.1016/S0167-8140(18)30999-X

[36] Bornstein MM, Horner K, Jacobs R. Use of cone beam computed tomography in implant dentistry: current concepts, indications and limitations for clinical practice and research. Periodontol. 2017;73(1):51–72. 10.1111/prd.12161. 10.1111/prd.12161 28000270

[37] Albrektsson T, Tengvall P, Amengual-Peñafiel L, Coli P, Kotsakis G. Cochran dlimplications of considering peri‐implant bone loss a disease, a narrative review. J Clin Implant Dent Relat Res. 2022;24(4):532–43. 10.1111/cid.13102. 10.1111/cid.13102 PMC9542069 35639515

[38] Nobuhiro Y, Yoshiki M, Masaru A, Guang H, Keiichi S. Effect of attachment type on load distribution to implant abutments and the residual ridge in mandibular implant-supported overdentures. J Dent Biomech. 2015;6(0):1. 10.1177/1758736015576009. 10.1177/1758736015576009 PMC4366420 25798201

[39] Turkyilmaz I, Tözüm TF, Tumer C, Ozbek, ENJJop. A 2-year clinical report of patients treated with two loading protocols for mandibular overdentures: early versus conventional loading. J Periodontol. 2006;77(12):1998–2004. 10.1902/jop.2006.060115. 17209784 10.1902/jop.2006.060115

[40] Aboelez MA, Elezz MGA, Abdraboh AE, Elsyad MAJCID, Research R. Angled ball and locator attachments for immediate loaded inclined implants used to retain maxillary overdentures: A cross over study of patient satisfaction and oral health related quality of life. Clin Implant Dent Relat Res. 2022;24(3):391–400. 10.1111/cid.13089. 35503746 10.1111/cid.13089

[41] Faty MA, Sabet ME, Thabet YGJIJP. A comparison of denture base retention and adaptation between CAD/CAM and conventional fabrication techniques. Int J Prosthodont. 2023;36(4):59–63. 10.11607/ijp.7193. 37699188 10.11607/ijp.7193

[42] Srinivasan M, Cantin Y, Mehl A, Gjengedal H, Müller F, Schimmel, MJCoi. CAD/CAM milled removable complete dentures: an in vitro evaluation of trueness. Clin Oral Investig. 2019;21(6):2007–19. 10.1007/s00784-016-1989-7. 10.1007/s00784-016-1989-7 27826696

[43] Kalberer N, Mehl A, Schimmel M, Müller F. Srinivasan MJTJopd.CAD-CAM milled versus rapidly prototyped (3D-printed) complete dentures: an in vitro evaluation of trueness. J Prosthet Dent. 2019;121(4):637–43. 10.1016/j.prosdent.2018.09.001. 30711292 10.1016/j.prosdent.2018.09.001

[44] Gad MM, Al-Thobity AM, Fouda SM. Flexural and surface properties of PMMA denture base material modified with thymoquinone as an antifungal agent. J Prosthodont. 2020;29:243–50. 10.1111/jopr.12967. 30178899 10.1111/jopr.12967

[45] Lee S, ∙ Hong SJ, ∙Pack J. Comparing accuracy of denture bases fabricated by injection molding, CAD-CAM milling and rapid prototyping method. J Adv Prosthodont. 2019;11:55–64. 10.4047/jap.2019.11.1.55. 30847050 10.4047/jap.2019.11.1.55 PMC6400705

[46] El-Shaheed NHL, Salama R, Faramawy AMG, Mostafa AZH. Tissue surface adaptation and clinical performance of CAD-CAM milled versus conventional implant-assisted mandibular overdenture. Int J Dent. 2023;11. 10.1155/2022/8220233. 10.1155/2022/8220233 PMC9225844 35756959

[47] Hsu CY, Yang TC, Wang TM, Lin LDJTJPD. Effects of fabrication techniques on denture base adaptation: an in vitro study. J Prosthet Dent. 2020;124(6):740–7. 10.1016/j.prosdent.2020.02.012. 32448642 10.1016/j.prosdent.2020.02.012

[48] Helal MA, Abdelrahim RA, Zeidan AAEJJP. Comparison of dimensional changes between CAD-CAM milled complete denture bases and 3D printed complete denture bases: an in vitro study. J Prosthodont. 2023;32(S1):11–9. 10.1111/jopr.13538. 35524633 10.1111/jopr.13538

[49] Chaturvedi S, Alqahtani NM, Al-Qarni MA, Alqahtani SM, Suleman G, Yaqoob A, Abdul Khader M, Elmahdi A, Chaturvedi M. Evaluation of the methods for determining accuracy of fit and precision of RPD framework in digital (3D printed, milled) and conventional RPDs - a systematic review. BMC Oral Health. 2024;24(1):1466. 10.1186/s12903-024-05262-x. 39633297 10.1186/s12903-024-05262-x PMC11619457

